# Comprehensive Analysis of Applicability Domains of QSPR Models for Chemical Reactions

**DOI:** 10.3390/ijms21155542

**Published:** 2020-08-03

**Authors:** Assima Rakhimbekova, Timur I. Madzhidov, Ramil I. Nugmanov, Timur R. Gimadiev, Igor I. Baskin, Alexandre Varnek

**Affiliations:** 1 A.M. Butlerov Institute of Chemistry, Kazan Federal University, 420008 Kazan, Russia; ARahimbekova@kpfu.ru (A.R.); RaINugmanov@kpfu.ru (R.I.N.); igbaskin@gmail.com (I.I.B.); 2 Institute for Chemical Reaction Design and Discovery, Hokkaido University, Sapporo 001-0021, Japan; gimadiev@icredd.hokudai.ac.jp; 3 Faculty of Physics, Moscow State University, 119234 Moscow, Russia; 4 Laboratory of Chemoinformatics, UMR 7140 CNRS, University of Strasbourg, 67000 Strasbourg, France

**Keywords:** applicability domain, Quantitative Reaction–Property Relationship, QSAR/QSPR, chemical reactions, chemoinformatics, machine learning, reaction mining

## Abstract

Nowadays, the problem of the model’s applicability domain (AD) definition is an active research topic in chemoinformatics. Although many various AD definitions for the models predicting properties of molecules (Quantitative Structure-Activity/Property Relationship (QSAR/QSPR) models) were described in the literature, no one for chemical reactions (Quantitative Reaction-Property Relationships (QRPR)) has been reported to date. The point is that a chemical reaction is a much more complex object than an individual molecule, and its yield, thermodynamic and kinetic characteristics depend not only on the structures of reactants and products but also on experimental conditions. The QRPR models’ performance largely depends on the way that chemical transformation is encoded. In this study, various AD definition methods extensively used in QSAR/QSPR studies of individual molecules, as well as several novel approaches suggested in this work for reactions, were benchmarked on several reaction datasets. The ability to exclude wrong reaction types, increase coverage, improve the model performance and detect Y-outliers were tested. As a result, several “best” AD definitions for the QRPR models predicting reaction characteristics have been revealed and tested on a previously published external dataset with a clear AD definition problem.

## 1. Introduction

Nowadays, Quantitative Structure-Activity/Property Relationship (QSAR/QSPR) models are widely used for predicting the properties of chemical compounds [1,2]. At the same time, QSAR/QSPR models are not universal, and their predictive performance highly depends on their similarity to training examples [3,4]. The Applicability Domain (AD) of a QSAR/QSPR model highlights a part of the chemical space containing those compounds for which the model is supposed to provide reliable predictions [5,6]. Therefore, the problem of defining the AD of a model is closely related to the problem of assessing the reliability of its predictions. According to the Organization of Economic Co-operation and Development (OECD) principles, QSAR/QSPR models should have “defined an applicability domain” [7].

The problem of AD estimation is an active research field in chemoinformatics. Many methods have been developed to estimate the reliability of the predictions provided by QSAR/QSPR models, see reviews [1,2,3,4,5,8,9,10]. They are based on different hypotheses, algorithms, concepts and often consider different types of information [9,10,11]. Such a wide variety of methods suggests that the term “applicability domain of models” has several meanings. For example, Hanser et al. [12] highlighted three aspects of AD definition: applicability, reliability, and decidability. The applicability of the model reflects that the test set is drawn from the same distribution as the training set. The reliability of the model characterizes the density of data around the test set object, which should be high enough. Decidability reflects the confidence of prediction. In this case, predictions made using QSAR/QSPR models can be trusted only if the requirements of all three types of criteria are met.

Algorithms to assess the AD of QSAR/QSPR models must find a trade-off between the coverage of chemical compounds (or, generally, chemical objects, which can also be mixtures of chemical compounds, chemical reactions, etc.) that are considered belonging to the model’s AD and the quality of predicting their activities/properties by the QSAR/QSPR model. In accordance with the terminology adopted in some fields of mathematical statistics and machine learning, the objects inside AD are denoted in this paper by the term X-inliers, and outside it—X-outliers. X-inliers and X-outliers are opposed to Y-inliers and Y-outliers, which correspond to the objects for which properties are well or poorly predicted by the model. The percentage of X-inliers in test data is the coverage of the model. At one extreme, all objects are considered as X-inliers and therefore a model can universally be applied to any test object with the same reliability. In this case, the coverage is 100%. This, obviously, contradicts the theory and practice of applying QSAR/QSPR models, which in the general case cannot be considered universal laws of nature. At the opposite extreme, only the training data are considered as X-inliers, while all other objects are considered as X-outliers. Therefore, the coverage is 0%. Such models are obviously useless in practice because they cannot be used to predict the properties of new objects. Therefore, AD for QSAR/QSPR models that are useful in practice should be between these two extremes. In this article, we find optimal AD definitions by maximizing certain AD performance metrics.

Nowadays the growing attention is attracted to chemical reactions as objects of QSAR/QSPR-like modelling [13,14,15,16,17]. Although numerous approaches are considered in the literature to assess the AD for the models predicting the properties of chemical compounds [3], ADs have almost never been applied to the models predicting the characteristics of chemical reactions, and the problem of AD definition for chemical reactions has never been discussed in the literature. Recently, several models for predicting the following numerical characteristics of chemical reactions have been published: rate constants [17,18,19,20,21,22], tautomeric equilibrium constants [23], reaction conditions [24], etc. However, the only attempt to determine AD by using a combination of fragment control, bounding box and “reaction signature control” was made in our previous article [23], which did not result in any reasonable recommendation because of either a low ability to discard Y-outliers or low data coverage.

It is much more difficult to define AD for the models aimed at predicting different characteristics of chemical reactions in comparison with standard QSAR/QSPR models dealing with the properties of chemical compounds because it is necessary to consider several important factors (reaction representation, conditions, reaction type, atom-to-atom mapping, etc.) that are specific to chemical reactions. The fact that different types of chemical reactions are characterized by specific mechanisms, and therefore a model trained on one reaction type (we call it in this paper the native reaction type) cannot predict the characteristics of the reactions belonging to other types reliably, is also important.

The major goal of this paper is to find the best AD definition approaches suitable for the models predicting quantitative characteristics of chemical reactions (Quantitative Reaction-Property models (QRPR). For this purpose, different commonly used AD definition methods and their modifications were benchmarked on four QRPR models. As the performance metrics, we consider the ability to: (i) detect reactions belonging to non-native types; (ii) improve model performance within AD; (iii) filter out Y-outliers; (iv) cover as many objects as possible. We find the optimal values of AD thresholds and hyperparameters using a special procedure including both internal and external cross-validation. 

## 2. Computational Approaches

### 2.1. Approaches for Defining Applicability Domain of QRPR Model

AD definition approaches can be considered as binary classifiers returning *True* for X-inliers (within AD) and *False* for X-outliers (outside AD). In this work, AD definition approaches are conditionally divided into two groups: (1) universal and (2) applied Machine Learning (ML) method-dependent.

The first group includes methods that can be used on top of QRPR models implemented by any machine learning method (denoted as Universal AD), and the second one includes methods that are integral parts of the machine learning (ML) methods implementing the QRPR models (denoted as ML-dependent AD). Such models not only predict reaction property values but also estimate the confidence of prediction, so the reactions with high confidence of prediction are within AD. Here, we only consider regression models and corresponding ADs. 

#### 2.1.1. Universal Applicability Domain Definition Approaches

Universal AD definition methods can be used on top of QRPR models, which can be implemented by any suitable machine learning method. In this study, the Random Forest Regressor (see below) is used for this purpose. Some AD definition methods (Leverage, Nearest Neighbors approach, One-Class SVM, Two-Class Y-inlier/Y-outlier classifier) return a continuous value indicating the reliability of prediction. When using these methods, it is necessary to choose a threshold for such a value, and for some of them, the values of other adjustable hyperparameters. Such methods correspond to the reliability aspect of AD definition according to Hanser et al. [12]. Some other methods (Bounding Box, Fragment Control, Reaction Type Control) give an answer as to whether a test object is within AD or not. Bounding Box and Fragment Control does not have any adjustable hyperparameter, whereas Reaction Type Control has one (neighborhood radius, see below). Such methods correspond to the applicability aspect of AD definition according to Hanser et al. [12].

**Leverage**. This method is based on the Mahalanobis distance to the centre of the training-set distribution. The leverage *h* of a chemical reaction is calculated based on the “hat” matrix as *h* = *(x_i_^T^(X^T^X)^−1^x_i_),* where *X* is the training-set descriptor matrix, and *x_i_* is the descriptor vector for the reaction *i*. The leverage threshold is usually defined as h* = 3*(M + 1)/N, where *M* is the number of descriptors and *N* is the number of training examples. Chemical reactions with leverage values *h* > h* are considered to be chemically different from the training set reactions, so they are marked as X-outliers [3,4]. This approach is denoted hereafter as **Leverage.** The drawback of it is the absence of strict rules for choosing the threshold h* [25]. As an alternative, an optimal threshold value h* can be found using an internal cross-validation procedure by maximizing some AD performance metrics. This method is denoted hereafter as **Lev_cv**. **Leverage** does not have internal hyperparameters, while **Lev_cv** has one—the optimal threshold that needs to be adjusted.

**Nearest Neighbours approach** (denoted as **Z-kNN**). This AD definition is based on the distance(s) between a current reaction and the closest training-set reaction(s). Usually, one nearest neighbour is considered (*k* = 1). If the distance is not within the user-defined threshold, then the prediction is considered unreliable and the reaction is considered as an X-outlier. The threshold value is commonly taken as *Dc = Zσ + <y>,* where *<y>* is the average and *σ* is the standard deviation of the Euclidean distances between nearest neighbours in the training set, *Z* is an empirical parameter to control the significance level, with the recommended value of 0.5 [3,4,25]. Such a method is denoted as **Z-1NN**. An optimal threshold can be found using an internal cross-validation procedure by maximizing some AD performance metrics. In this case, the method is denoted as **Z-1NN_cv**.

**One-Class Support Vector Machine** (denoted as **1-SVM**). The one-class Support Vector Machine method reveals highly populated zones in descriptor space by maximizing the distance between a separating hyperplane and the origin in the feature space implicitly defined by some Mercers’ kernel. The decision function of such model returns (+1) for the reactions which fall into highly populated zones (within AD, i.e., X-inliers) and (−1) for the reactions outside of AD (X-outliers) [9,26]. **1-SVM** models were built in this study using the scikit-learn library [27]. The method requires the fitting of two hyperparameters: *nu* (which defines the upper bound percentage of errors and lower bound percentage of support vectors) and *gamma* (parameter of RBF kernel which is used), the optimal values of which can be found in cross-validation (see Table S1). Other hyperparameters were set to default values.

**Two-Class Y-inlier/Y-outlier Classifier** (denoted as **2CC**). In this case, a binary classifier learns to distinguish Y-inliers from Y-outliers. First, QRPR models are built to predict quantitative characteristics of chemical reactions. Chemical reactions with a higher prediction error estimated in cross-validation (more than 3× RMSE) are labelled as Y-outliers, while the remaining reactions are labelled as Y-inliers. After that, a binary classification model is trained to discriminate between them and provide a confidence score that a given reaction is a Y-inlier for the corresponding QRPR model. Although this method seems quite straightforward, we have not found its application in the literature. Unfortunately, this method cannot be applied if there are no or too few Y-outliers. In this study, Random Forest Classifier, implemented in scikit-learn library [27], was used for building the binary classification model. The method requires setting the values of two hyperparameters: *max_features* (the values of features selected upon tree branching) and probability threshold p* (see Table S1). If the predicted probability of belonging to the Y-inliers is greater than p*, the prediction of reaction characteristics by the QRPR model for it is considered reliable (within AD, or X-inlier). Other hyperparameters of the Random Forest Classifier were set to defaults, except the number of decision trees in Random Forest Classifier, which was set to 500.

**Bounding Box** (denoted as **BB**). This approach defines AD as a D-dimensional hypercube with each edge spanning the range between the minimum and maximum values of the corresponding descriptor. If at least one descriptor for a given reaction is out of the range defined by the minimum and maximum values of the training set examples, the reaction is considered outside of the AD of the corresponding QRPR model [3,4]. The approach does not have adjustable hyperparameters.

**Figure 1:**
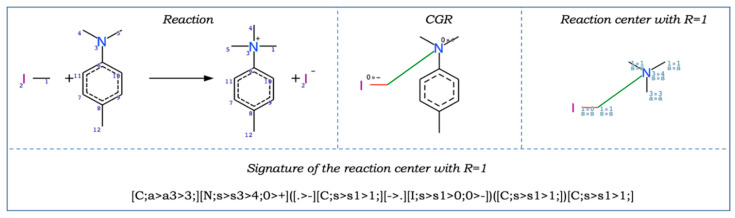
A chemical reaction (top left), its Condensed Graph of Reaction (CGR) (top centre), and the reaction centre with *R* = 1 (top right). Colour lines on CGR represent dynamic bonds. Indices “0 >> −” and “0 >> +” on CGR mean that atomic charges are lowered from 0 to −1 and increased from 0 to +1, respectively. Indices “1 >> 0” and 1 >> 1” on the reaction center mean changes in the number of neighbors, letters—hybridization changes (“s”—sp^3^, “a”—atom is in the aromatic ring). The signature of the reaction center with *R* = 1 is shown.

**Fragment Control** (denoted as **FC**). In this case, if a Condensed Graph of Reaction [28,29] (Figure 1) representing a given reaction has fragments (subgraphs) missing in the training set, then it is considered to be an X-outlier (out of AD) whenever the corresponding QRPR model is applied [8,30]. **FC** can formally be considered as a special case of Bounding Box for fragment descriptors. This method does not have adjustable parameters.

**Reaction Type Control** (denoted as **RTC_cv**). This method was proposed for the first time in our previous publication [16] for predicting kinetics parameters. If the reaction centre of a chemical reaction is absent in the reactions in the training set, it is considered out of AD (X-outlier). Reaction centre is detected using reaction signatures [31]. Signature creation includes (1) representation of a chemical reaction as a Condensed Graph of Reaction (CGR, Figure 1, top center), (2) highlighting one or more reaction centers which are identified as a set of adjacent dynamic atoms and bonds on the CGR and (3) considering atoms in neighborhood with radius *R* for each of the reaction centers (Figure 1, top right), (4) introducing canonical numbering of atoms of the reaction center with its neighborhood using an algorithm similar to the Morgan algorithm, (5) the signature is encoded by SMILES-like canonical string generated by CGRtools library (Figure 1) [31]. For every atom, hybridization, the number of adjacent heavy atoms and the symbol of chemical element are encoded in the signature. In order to distinguish whether the aromatic cycle is a part of the reaction center or its closest substituent, we introduced a separate type of hybridization for aromatic atoms. Thus, sp^3^, sp^2^, sp hybridizations for aliphatic atoms and “aromatic” hybridizations were used. The signature includes atomic labels both on the reaction centre itself and its neighbourhood. The neighborhood radius *R* is a hyperparameter of the method. If the radius is set to 0, the reaction signature includes only atoms of the reaction centre. Since it is necessary to select the hyperparameter, this method is denoted as **RTC_cv**.

#### 2.1.2. ML-Dependent Applicability Domain Definition Approaches

**The variance in the prediction density given by Gaussian Process Regression model** (denoted as **GPR-AD**). Gaussian Process Regression (GPR) assumes that the joint distribution of a real-valued property of chemical reactions and their descriptors is multivariate normal (Gaussian) with the elements of its covariance matrix computed by means of special covariance functions (kernels). For every reaction, a GPR model produces a posterior conditional distribution (so-called prediction density) of the reaction property given the vector of reaction descriptors. The prediction density has normal (Gaussian) distribution with the mean corresponding to the predicted value of the property and the variance corresponding to prediction confidence [32]. If the variance is greater than a predefined threshold σ*, the chemical reaction is considered as X-outlier (out of AD). This AD definition method is denoted as **GPR-AD.** The GPR implementation in scikit-learn library was used [27]. The method requires adjustment of three hyperparameter—*alpha*, which stands for the noise level (also acts as regularization of the model), the parameter *gamma* of the RBF kernel which represents the covariance function (see Table S1), and variance threshold σ*. The optimal values of hyperparameters are determined using internal cross-validation. Other hyperparameters of Gaussian Processes are set by default.

**The variance in predictions made by an ensemble of QRPR models.** The variance in predictions made by an ensemble of QSAR/QSPR models is often applied as a score for determining their AD [5,6]. In this study, we extend this to QRPR models. Here, we consider a chemical reaction to be within AD (X-inlier) if the variance in the property values predicted by the ensemble of models is less than a given threshold σ*. The optimal value for σ* can be found using internal cross-validation procedure by maximizing an AD quality metrics (see below). One of the approaches to estimate the prediction variance needed for this purpose is to build a QRPR model on the whole training set using the Random Forest Regression (RFR) machine learning method, which provides the mean (which is considered as a predicted value of the reaction property) and the variance in predictions (which is considered as a measure of prediction confidence) made by individual Random Trees individual models. This approach is denoted hereafter as **RFR_VAR**. In this study, a modified version of the Random Forest Regression method (RFR, 500 trees) implemented in scikit-learn library [27] was used.

#### 2.1.3. Other AD Definition Approaches

**Zero and perfect models.** Zero models can be suggested to compare different AD definitions with some simple rules. Zero models include “optimistic” AD definitions assuming that all reactions are within AD (denoted as **OZ**), while the “pessimistic” AD definition assumes that all reactions are out of AD (denoted as **PZ**). In addition to zero models, the “perfect” AD definition can be proposed for comparison. The **“Perfect AD model”** assumes that all Y-inliers are X-inliers (i.e., for all reactions within AD, absolute property prediction error is lower than 3× RMSE), and all Y-outliers are X-outliers (i.e., for all reactions outside AD, absolute property prediction error is higher than 3× RMSE).

### 2.2. AD Performance Metrics

To build the best AD definition models, it is necessary to optimize their thresholds and (hyper) parameters by maximizing some AD performance metric. The following metrics are considered in this study:

(i) **∆R^2^_AD**—the difference between the coefficient of determination for property prediction (R**^2^**) for reactions within AD and the corresponding value for all datapoints. Positive values of this metric indicate the improvement in the quantitative property prediction within AD;
(ii) **OIR** (Out and in RMSE)—the difference between RMSE of property prediction for reactions outside AD (denoted as RMSE_OUT_) and within AD (denoted as RMSE_IN_). The metric was first proposed by Sahigata et al. [25]. Negative values indicate that the reactions detected as X-outliers (outside AD) are predicted better than X-inliers (within AD), thus highlighting some possible drawbacks in the definition of interpolation space. Its positive values indicate the improvement in the property prediction within AD. If no reactions are left inside or outside AD, then **OIR** is considered equal to 0;
(iii) The **Outliers Criterion** metric shows how well AD definition detects Y-outliers. First, property prediction errors are estimated in cross-validation for all reactions in a dataset. The reactions for which the absolute prediction error is higher than 3× RMSE are identified as Y-outliers, while the rest are considered as Y-inliers. Y-outliers (poorly predicted) that are predicted by AD definition as X-outliers (outside AD) are called true outliers (TO), while Y-inliers predicted by AD definition as X-inliers (within AD) are called true inliers (TI). False outliers (FO) are Y-inliers that are wrongly predicted by the AD definition as X-outliers, while false inliers (FI) are Y-outliers that are wrongly predicted by the AD definition as X-inliers. Then, the quality of outliers/inliers determination can be assessed using an analogue of the balanced accuracy and denoted as **OD** (Outliers Detection). **OD** = $\left(\frac{TO}{TO+FI}+\frac{TI}{TI+FO}\right)/2$. In the “**Perfect AD model**”, the value of this parameter approaches the maximum possible value of 1;
(iv) **Area Under the ROC Curve** (**AUC_AD)**. This metric shows how well an AD definition can rank predictions made by the corresponding QRPR model from most reliable to least reliable. Reactions that are considered by AD definition as X-inliers (within AD) are considered as the positive class, while X-outliers are considered as the negative class. The absolute error of property prediction made by the corresponding QRPR model for a given reaction is used as a threshold for ROC curve construction. Thus, if the Area Under Curve (AUC) is large (close to 1), X-outliers are at the top of the list, sorted in descending order according to the error of prediction.

We have found in the initial stages of the study that **∆R^2^_AD** and **AUC_AD** are not suitable objective functions for optimizing thresholds and hyperparameters of AD definition models, because they often lead either to very small coverage with high accuracy (when only a few points very similar to training examples are left within AD) or, vice versa, high coverage and low accuracy (because a very small number of excluded examples can only slightly improve the accuracy of the whole dataset). The **∆R^2^_AD** and **AUC_AD** metrics appear not to be able to distinguish between these two modes. Since the resulting AD models selected using these metrics had very different coverage, their comparison was difficult. For this reason, we refused to use these metrics to benchmark different AD definition models.

### 2.3. Model Building and Validation

**Building and validation of QRPR models.** For Universal AD definition approaches, only the Random Forest Regression (RFR) was used for building QRPR models. For Machine Learning method-dependent (ML-dependent) AD definition approaches, both RFR and GPR machine learning methods were used for this purpose, see above.

**Figure 2:**
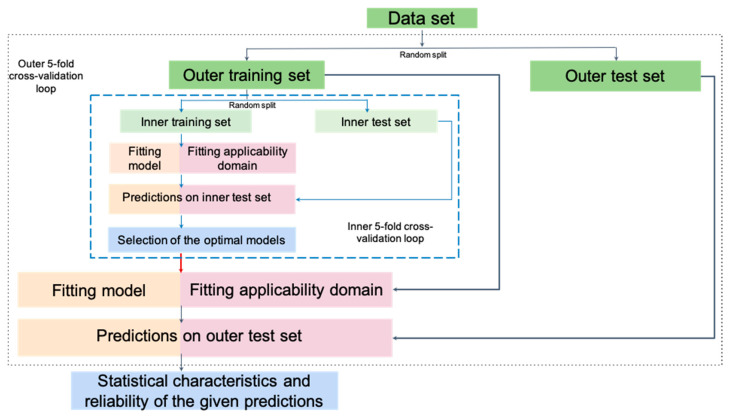
The procedure for selecting the hyperparameters of Quantitative Reaction–Property Relationships (QRPR) and the AD definition models.

In this study, implementations of the RFR and GPR methods were taken from the scikit-learn library [27], and RFR was modified to return variance of prediction. The number of trees in RFR was set to 500, while the only tuneable hyperparameter was the number of features selected upon tree branching (*max_features*). Other hyperparameters in RFR were set to default values. For GPR models, hyperparameters of noise level, *alpha*, and RBF kernel’s *gamma* values were adjusted (see Supporting Materials (Table S1) for grid values). To obtain a reliable assessment of predictive performance and avoid overfitting, the nested cross-validation procedure [33] was used. For each training/test split in the outer loop, the hyperparameters of RFR and GPR models were tuned using grid search by minimizing the averaged RMSE of prediction (without AD application) estimated in the inner cross-validation loop on the outer training set, and the optimal models with the tuned hyperparameters were used to predict reaction properties on the outer test set, Figure 2.

**Building and validation of AD definition models.** Universal and ML-dependent AD definition methods, both with and without hyperparameters, are presented in Table 1. Since the hyperparameters of some AD definition methods need to be tuned, the above-mentioned nested cross-validation procedure was used, in which the inner 5-fold cross-validation loop was used for hyperparameter selection, whereas the outer 5-fold cross-validation loop was used for assessing predictive performance.

For AD definition approaches which do not require hyperparameter selection (Table 1), a regression QRPR model (RFR or GPR) and an AD definition model were built on the outer loop training set selected in each split of external cross-validation, and both the property and applicability domain membership (within AD or out of AD) for the external test set were predicted.

All hyperparameters of both QRPR and AD definition models were selected for each training/test split in the outer cross-validation loop by maximizing the **OIR** or **OD** metrics computed for the outer training set using the inner cross-validation loop (Figure 2). The selected hyperparameters were used to rebuild models on the outer training set (Figure 2), which were further used to predict reaction property and AD membership (within AD or out of AD) on the corresponding outer test set. After the completion of the outer loop, all values (predicted value and AD membership) predicted on individual outer test sets were merged, and the predictive performances of QRPR models with AD were assessed.

Consider the abbreviations used hereafter for denoting AD definition methods and the corresponding models. The name, in this case, consists of two components that characterize first the basic AD method applied and, second, the metric used for tuning its hyperparameter(s) (if needed). For example, the abbreviation **Z-1NN_cv/OD** means that the hyperparameters of the **Z-1NN_cv** AD definition methods were tuned by maximizing the **OD** AD performance metric.

In this study, 14 Universal AD definition methods are considered, including two methods without hyperparameters (**BB, FC)**, two methods for which only recommended default values hyperparameters were taken (**Leverage, Z-1NN**), five methods for which hyperparameters were tuned when building models (**1-SVM, RTC_cv, Z-1NN_cv, Leverage_cv, 2CC**), and two ML-dependent AD definition methods (**RFR_VAR, GPR-AD**). The latter two hyperparameters were optimized using either **OIR** or **OD** metrics. Two Zero Models (“optimistic” and “pessimistic” zero models) and “**Perfect AD Model**” were applied for comparison purpose. In total, 21 AD definition methods were benchmarked.

### 2.4. Data Sets and Descriptors

Four datasets for the following types of reactions were used in this study: bimolecular nucleophilic substitution (S_N_2) [16], bimolecular elimination (E2) [21], Diels-Alder reactions (DA) [22] and tautomerization (Tautomerization) [23]. These datasets were collected in our previous studies.

Chemical transformations were encoded by Condensed Graphs of Reaction (CGRs) (Figure 1) [29,31]. ISIDA fragment descriptors were generated for the CGRs using the ISIDA Fragmentor [30] program. ISIDA fragment descriptors represent subgraphs of different topologies and sizes. Each subgraph is considered as a descriptor, whereas its occurrence in a molecule is the descriptor’s value. In this study, sequences of atoms and bonds (subgraphs) were considered. The number of atoms in a fragment varies from 2 to 4, which was often one of the best fragmentation schemes in our previous studies [16,21,22,23]. Each solvent was described by 15 descriptors that represent polarity, polarizability, H-acidity and basicity: Catalan SPP [34], SA [35], and SB constants [34], Camlet–Taft constants α [36], β [37], and π* [38], four functions of the dielectric constant, three functions of the refractive index, as described in the paper [39]. The latter seven descriptors reflect the polarity and polarizability of the bulk of the solvent. The inverse absolute temperature, 1/T (in Kelvin degrees) was also used as a descriptor of temperature. Since some of the solvents used in this study are water-organic mixtures, the molar ratio of organic solvent was used as descriptor as well (100% for pure solvent). Descriptor vector for each reaction resulted from concatenation of structural descriptors and parameters describing experimental conditions (solvent and temperature).

All descriptors were normalized to zero mean and unit variance. In the case of GPR models, both descriptors and property values were normalized, because this provided better predictive performance. In the latter case, the performance of the models was estimated after rescaling the predicted values back to the initial range. The performances of GPR and RFR models for all datasets are reported in Table 2.

## 3. Results and Discussion

A systematic comparison of different AD definition methods was done using four datasets of reactions (S_N_2, E2, DA, Tautomerization). All results of benchmarking are collected in the Tables S2–S8. Hereafter, a brief comparison of different AD definitions with respect to the benchmarking criteria given in Section 2.2 is discussed. 

### 3.1. Detection of a Reaction Type Missing in the Training Set

An important criterion for the efficiency of AD definition methods applied to reactions is that a chemical reaction belonging to the type missing in the training set must be considered as an X-outlier (out of AD), while the reactions that belong to the type used for model building (hereafter called native reaction type) should be mostly attributed as X-inliers (within AD). This goal can be achieved algorithmically by applying Fragment Control (**FC**) or Reaction Type Control (**RTC_cv**, which is based on the analysis of the most complete representation of CGR in the form of a graph).

**Figure 3:**
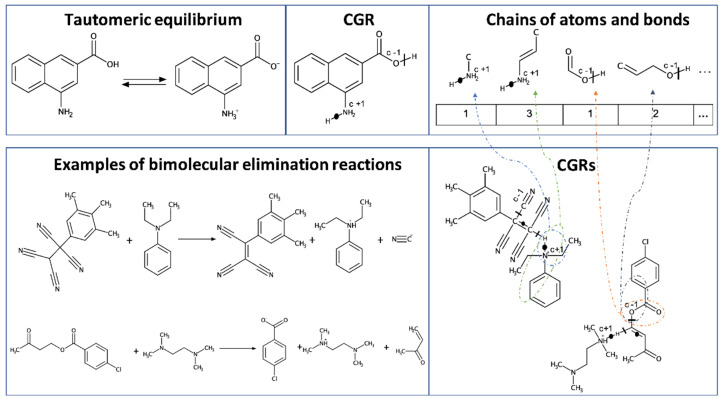
Fragment Control considers tautomerization reactions (presented at the top) as belonging to the AD defined for the E2 reactions (bottom) because all fragments of the CGRs of tautomerization reactions (top right) are present in the CGRs of the reactions contained in the E2 dataset. CGRs are shown to the right of the corresponding reaction.

Therefore, we have built AD definition models (**RTC_cv, FC**) on each of the four datasets and applied it to the other three datasets. Reaction Type Control correctly identify non-native reactions as X-outliers in 100% of cases. Fragment Control works correctly in 99% of cases by excluding from AD non-native reactions. However, in some cases, it can make mistakes by including tautomerization reactions into the AD defined for S_N_2 and E2 datasets. This can be explained by the fact that some tautomerization reactions belonging to the zwitterion-neutral molecule tautomerism have no dynamic bonds in their CGRs, and therefore no fragments with dynamic bonds can be generated. As a result, such tautomerization reactions can pass the Fragment Control filter (Figure 3).

Thus, the **FC** method, from which one would expect that it can cope well with the detection of a non-native reaction type through the explicit use of structural information, in some cases does not work correctly. Since it is an essential criterion for defining AD for reactions, **FC** cannot be considered as robust approach for detection of novel reaction type. Nonetheless, it can still be recommended to be applied to QSAR/QSPR models for molecular data. The previously proposed approach of Horvath et al. [40] uses similar combinations of Fragment Control with confidence-based approaches.

Based on the requirement that correct AD definition methods should identify non-native reaction types are X-outliers (out of AD), we have assumed that **RTC_cv** should be involved in defining AD for QRPR models. Reaction Type Control corresponds to the applicability aspect of AD definition according to Hanser et al. [12]. The **RTC_cv**, however, does not consider either local data density in chemical space, which corresponds to the reliability aspect of AD definition, nor decidability reflecting prediction confidence. Thus, Reaction Type Control should be used in combination with other AD definitions, which comprise composite AD definitions. The analysis of the ability of **RTC_cv** to filter out non-native reaction types has shown that it works correctly with non-native reactions for any neighbourhood radius (from 0 to 10). Hereafter, Reaction Type Control with the first neighbourhood is used as the default method (denoted as **RTC1**) as least restrictive but still working well with novel reaction types.

The rest of the paper deals only with combinations of **RTC1** with other definition methods. In this case, a chemical reaction is considered as X-inlier if it is X-inlier in both AD definition models. Such composite methods/models are denoted hereafter as BB*, FC*, 2CC*, Leverage*, Lev_cv*, Z-1NN*, Z-1NN_cv*, 1-SVM* (among Universal ADs), RFR_VAR*, GPR-AD* (ML-dependent ADs), where * means combination with **RTC1.**

Since **RTC1** correctly identify non-native reactions as X-outliers, all composite approaches detect non-native reaction types in 100% of cases.

### 3.2. Coverage

AD definitions are balanced between the coverage and the predictive performance. It is desirable that a QRPR model be applicable to the largest possible data space. Therefore, the coverage provided by AD definitions should be as large as possible. The values of coverage for all datasets for composite AD definition methods are presented in Table 3 (even though only composite AD definition methods are discussed in the paper, Table S2 contain data for all AD definitions (not only the composite ones)). “**Perfect AD model**” had coverage about 98–99%, **RTC1**—about 93–98% for all four datasets. The latter is caused by presence of some portion of reactions that highly differ from the rest of the dataset.

The coverage provided by most AD definition models optimized using the **OD** metric ranges from 0.70 to 0.95, except 1-SVM*/OD. For the latter, the coverage ranges from 0.29 to 0.68. The coverage for the AD definition models optimized using the **OIR** metric is larger, with the average value ranging from 0.94 to 1.0. AD definition models without hyperparameters are characterized by high coverage (0.91–0.99).

### 3.3. Detection of Y-outliers

The detection of potential Y-outliers, i.e., chemical objects with high property prediction errors, is the main goal of applying AD definition models. In this study, the ability to detect Y-outliers is characterized by an analogue of balanced accuracy (shorthand **OD**), as described in Section 2.2. This measure is dominated by the precision of Y-outlier detection, because typically the number of Y-inliers is much greater, and therefore the true share of Y-inliers is close to 1.

The values of **OD** estimated using the outer loop of the nested cross-validation, known also as “external cross-validation” [5], for all AD definition models are presented in Table 3 (results for all datasets for individual ADs are presented in Table S2). The balanced accuracy values for **Zero AD Models** and “**Perfect AD model**” are 0.5 and 1.0, respectively, as follows from their definition. As one might expect, AD definition models with hyperparameters tuned to maximize **OD** detect Y-outliers much better (higher values of **OD** on “external cross-validation”) than the AD definition models without hyperparameters or with hyperparameters tuned to maximize **OIR**, which tend to underestimate the number of Y-outliers.

As follows from the results presented in Table 3, 2CC*/OD, GPR*/OD, RFR_VAR*/OD are the best AD definition methods for Y-outlier detection. For some datasets, Z_1NN_cv*/OD and Lev_cv*/OD also show good performance of Y-outlier detection.

### 3.4. Enhancement of Prediction Quality

The practical value of using AD in QRPR is the ability to identify reactions with a potentially large error of property prediction (Y-outliers) and their exclusion from the consideration. Thus, AD definition models should guarantee that reactions outside AD (X-outliers) have, on average, higher property prediction errors than those within AD (X-inliers). The metric **OIR** (see Section 2.2) reflects the difference in prediction error between reactions outside of AD and within it. The positive values of **OIR** indicate the ability of an AD definition model to detect Y-outliers end enhance the prediction quality by excluding them from consideration.

The values of **OIR** estimated in the same way as **OD** for AD definition models are presented in Table 3 and Table S2 for individual ADs. The AD definition models with hyperparameters tuned by **OIR** maximization expectedly lead to higher values of **OIR** estimated using “external cross-validation” (actually, the outer loop of the nested cross-validation) than the AD definition models optimized using **OD**. Nonetheless, almost all AD definition models are characterized by a positive value of the **OIR** metric, substantially better than for zero models, even if the **OD** metric was used for tuning hyperparameters.

Meanwhile, in terms of practical utility, it is important to know how the exclusion of the reactions identified by an AD definition model as X-outliers affects the determination coefficient (R^2^) of the corresponding QRPR model. The AD definition models that select X-inliers with the highest R^2^ on the external test are the most interesting from the practical point of view. To find out, we have computed the ∆**R^2^_AD** metric for all AD definition models. Its values are presented in Table 3 and Table S2 for individual ADs. Surprisingly, the improvement in the R^2^ appears not to be high, and there is no correlation between the values of **∆R^2^_AD** and **OIR**. This may be because when using **OIR** as a metric for tuning hyperparameters, too few datapoints are identified as X-outliers, so their exclusion cannot lead to significant improvements in R^2^. 

At the same time, AD definition models optimized by **OD** appear to improve more significantly the value of R^2^ of QRPR models after the exclusion of X-outliers. Surprisingly, several AD definition models with hyperparameters tuned by maximizing **OD** (RFR_VAR*/OD, 2CC*/OD, GPR-AD*/OD) improve R^2^ so significantly that ∆**R^2^_AD** for them are even better than for “**Perfect AD model**” This is caused by the fact that the former models excluded the significant number of worst-predicted datapoints, allowing further improvement in the R^2^ of the model in comparison with a “**Perfect AD model**”. The mentioned AD models exclude not only Y-outliers but also some Y-inliers which, however, are predicted with high error (but below 3∙RMSE). Thus, R^2^ on the external set improved better than in the “**Perfect AD model**”, which excludes only datapoints with prediction error higher than 3∙RMSE. Notice that the AD definition methods with hyperparameters tuned by maximizing **OD** generally have smaller coverage as compared to maximizing **OIR.**

### 3.5. Ranking Different AD Definition Methods

This study concerns four AD performance metrics, four datasets and 19 AD definition methods (results for all datasets are presented in Tables S3–S7. For each characteristic (coverage, the ability to improve performance of QRPR model and to detect Y-outliers) and for every dataset, we have assigned a zero penalty to those AD definition models for which the value of the characteristic is in the top 50% of the best values for it, and a penalty equal to one in the opposite case. For the non-native reaction type recognition criterion, a penalty equal to one was assigned if at least one non-native reaction was predicted as X-inlier (within AD). The results obtained after ranging the sums of all penalties are presented in Tables S3–S6. The best five AD definition methods for each dataset are presented in Table 4.

The first thing that is noticeable when considering this table is that “**Perfect AD model”** is always at the top of each ranked list, meaning that it is indeed a good approximation to the perfect AD definition. The most important conclusion following from the analysis of this table is that there is no single universally best AD definition method, so each dataset is characterized by its own list of preferred methods.

Although there is no method that would be best for each individual dataset, nevertheless, all methods can be ranked by how often they get to the top of the lists for different datasets. To obtain such lists, we have summed the ranks for each method over all datasets. It has been found that the “**Perfect AD model”** is, expectedly, the first method in the ranked list again followed by ML-dependent AD definition methods RFR_VAR*/OIR, GPR-AD*/OIR and Universal AD definition methods 2CC*/OIR. These methods are characterized by high coverage; they correctly detect Y-outliers, always detect correctly non-native reactions, and the performance of QRPR models is always improved after the exclusion of X-outliers. Therefore, they show good results for all datasets and thus can be recommended in most cases. 

### 3.6. Validating AD Definition Models on External Test Set

To test the selected AD definition methods, we took an external test set described in our previous publication [16], in which we first faced the problem of AD definitions for reactions. In the original paper [16], QRPR models were built for the rate constant of S_N_2 reactions, while the external test set contained 105 Menshutkin reactions. It was shown in that publication that the QRPR model predicted most reaction rate constants quite well, except one group of reactions involving a substituted phenylsulphonate leaving group, for which almost the same value of the rate constant was predicted by the model. It was shown that none of the ADs used in the original publication [16] (Fragment Control and the early version of Reaction Type Control with different neighbourhoods) can be recommended, because of either a low ability to discard the outliers or low data coverage.

The prediction performance of the Random Forest Regressor model (see Section 2.3) on whole test set (R^2^ = 0.60, RMSE = 0.84) is close to those of the SVR consensus models by Gimadiev et al. (R^2^ = 0.64, RMSE = 0.80) [16]. The above-discussed workflow for building regression and AD models were applied without changes. The ten best-ranked AD definition models (see Section 3.5) were trained on the data taken from the publication [16] and applied to the external test set. The statistical parameters of the models computed on the external test are presented in Table 5. Notice that simple approaches, which do not require any hyperparameter adjustment, such as RTC1 and BB*, are in the list of the best AD definitions as well.

**Figure 4:**
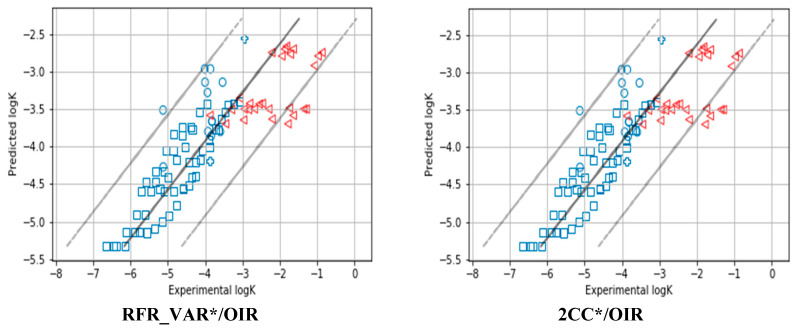

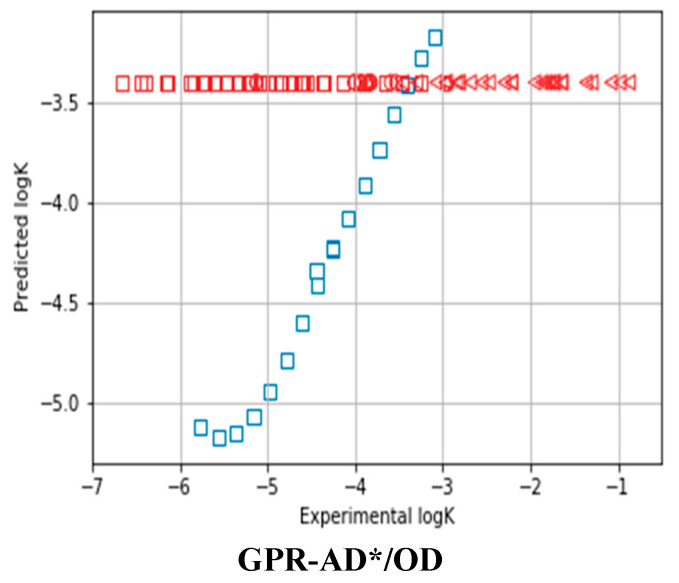
Validation of the model on the external test set: predicted vs. experimental logk. Blue datapoints are reactions within AD (X-inliers); red datapoints are outside AD (X-outliers). Reactions with different reaction centers are specified by different shapes of points: reactions with phenylsulphonate leaving group for which rate constants were predicted badly by ML methods are shown as triangles. Reactions with chlorine leaving group are shown as circles, bromine leaving group—as crosses, iodine leaving group—as squares.

Although an ML-dependent AD definition model GPR-AD*/OIR significantly improves the performance of QRPR model (R^2^), the coverage is only 17% of the dataset. One can notice (see Figure 4) that Gaussian Process Regressor almost perfectly predicts reaction rates for only 17% of reactions, but for the rest, the same value is predicted. All the latter points were identified as X-outliers (out of AD). It seems that the Gaussian Process method is more sensible to data novelty than Random Forest and predicts property well for only a small part of the test set reactions. However, it correctly assigns large variance of prediction for such poorly predicted test set objects, so they can be detected and filtered out. Therefore, if GPR fails to predict property for some objects, **GPR-AD** correctly identifies them as X-outliers. Thus, we can recommend using them together.

When using ML-dependent AD RFR_VAR*/OIR and Universal AD 2CC*/OIR, the highest coverage was observed—74%. The performance of the test set prediction was significantly better (RMSE = 0.64 and R^2^ = 0.66) than the observed model without AD. The inspection of Figure 4 shows that these two methods correctly identified reactions that were out of AD according to the manual analysis performed in paper [16]. Namely, the RFR_VAR*/OIR and 2CC*/OIR discarded only those reactions that contain a substituted phenylsulphonate leaving group which were predicted with the same values by Random Forest regressor (such reactions were labelled with triangles in Figure 4). It should be noted that **RFR_VAR/OIR** and **2CC/OIR** without **RTC1** do not identify any reaction with substituted phenylsulphonate leaving group as an X-outlier (out of AD); coverage was observed as 100% in this case (Table S8). A closer look at the prediction of RFR_VAR*/OIR and 2CC*/OIR composite AD definition methods shows that the **RTC1** AD definition method used in this study correctly identifies these reactions. **RTC1** has the same quality metrics as RFR_VAR*/OIR and 2CC*/OIR (see Line 8 in Table 5). Therefore, for this particular case, it is enough to use only **RTC1** to define AD.

Thus, for modelling the properties of chemical reactions, Reaction Type Control, as an approach for defining the applicability domain of the model works, strikingly well. By itself, Reaction Type Control is not at the top of the best methods for all characteristics (Table 3 and Table 4), however, it detects correctly not only non-native reactions but also structurally new reactions of native type as X-outliers (out of AD). Its combination with other AD definition methods can catch subtle effects in reaction datasets, and therefore the above-mentioned best three AD definition approaches perform really well on the tough case considered in paper [16], where X-outliers are very similar to the reactions in the training set.

## 4. Conclusions

In this paper, we have presented a workflow to compare and search for the best methods for defining the applicability domain (AD) of Quantitative Reaction–Property relationships (QRPR) models. However, the same technology can also be applied to QSAR/QSPR models for molecular data sets, except the consideration of reaction-specific ADs (Reaction Type Control and its combinations with other methods). The proposed methodology is quite flexible, so one can choose which AD definition method is preferable in each case.

In this study, different popular AD definition methods were benchmarked. Unlike some previous studies [9,10,11], here, special attention is paid to AD definition methods in application on regression models. AD prediction is considered in this study as a binary classification (inside/outside AD). The rationale behind the latter is that a chemist should be able to get a clear answer as to whether he/she can trust or ignore the prediction of the model. Based on this, quantitative metrics are proposed in this paper that assess the performance of AD definitions according to coverage, improvement in predictive performance using AD, ability to detect reactions with poorly predicted properties (Y-outliers) and the possibility of AD definition models to detect reactions of a type that does not present in the training set (non-native reactions). Two novel AD definition methods are proposed (**2CC, RTC_cv**). Two zero models (“optimistic” and “pessimistic”) and the “**Perfect AD model**” are proposed as baselines for comparison. The selection of optimal AD models is considered as a machine-learning hyperparameter selection problem, and thus internal cross-validation (actually, the inner loop in nested cross-validation) is applied to select optimal hyperparameters of AD definition. Four datasets of reactions are used for benchmark study. Different metrics, **OIR**, which was proposed earlier [25] and **OD**, which is proposed in the present publication, are used as objective functions to tune hyperparameters. **OIR** is shown in this paper to give models with higher coverage in comparison with **OD**, which gives stricter AD definitions. The latter leaves fewer datapoints within AD but provides much better predictions for them. The other metrics, including difference in R^2^ for points within AD and all of them, and AUC_AD as the ranking criterion, appear not to be suitable for tuning hyperparameters. 

In this study, it was suggested that some filters should be applied that reveal structural novelty directly prior to descriptor calculation (since descriptor vector calculation loses structural information). Among the latter, Reaction Type Control and Fragment Control can be noted. However, it is shown in this paper that, in some cases, Fragment Control fails to detect non-native reaction types (1% of reactions of non-native dataset), so its application as an AD definition method for reactions is undesirable. Nonetheless, it can still be recommended to be applied to QSAR/QSPR models for molecular data.

Based on the assumption that correct AD definition methods should identify non-native reaction types as X-outliers (out of AD), we concluded that **RTC_cv** should be involved in defining AD for QRPR models**.** The combination of **RTC_cv** with other AD definition methods is beneficial because additional AD definition methods can reveal subtle effects in data distribution, like distance of the data in the training set, data density or confidence. It is also shown that Reaction Type Control can detect not only non-native reactions but also structurally new reactions of native type as X-outliers (out of AD). The Reaction Type Control with the first neighbourhood can be offered as the recommended method in combination with other approaches (denoted as **RTC1**).

It has been demonstrated that most composite AD definition methods are quite good at detecting non-native reaction types, to improve the performance of QRPR models after exclusion of X-outliers, and to detect Y-outliers (reactions with high property prediction errors). The combined AD definition methods are shown in this paper to lead to much better model performance than individual AD definition methods (like **Leverage, z-kNN**). Based on a simple ranking scheme, it is shown in this paper that the best AD definition methods are combinations of **RTC1** with **GPR-AD** (Gaussian processes in which the variance of prediction density is used to estimate the AD of the model), with **RFR_VAR** (Random Forest in which the variance of predictions provided by individual trees is used to assess the confidence of predictions), and with the method **2CC** (put forward in this paper) based on the confidence of the two-class classification models. 

The best-selected AD definition methods were tested in this paper on a challenging task taken from paper [16], where reactions for which rate constants are predicted with high errors are similar to reactions in the training set. The best 10 composite AD definition methods provide rather different coverage in this case because the applied ML methods (Random Forest and Gaussian Process Regressors) are characterized by different ability to generalize to new reaction types and the use of **OD** as the objective function for maximization results in stricter AD definitions. Nonetheless, all of them detect Y-outliers (reactions with high property prediction errors) rather well. Thus, these methods, especially RFR_VAR*/OIR, 2CC*/OIR, and GPR-AD*/OD can be recommended to be used for chemical reactions, showing good performance on all datasets under study.

All software tools developed in this study to define AD are freely available on GitHub (https://github.com/cimm-kzn/CIMtools). Tutorial on AD definition and validation is available on https://github.com/cimm-kzn/CIMtools/tree/master/doc/tutorial/. The datasets used in this study are published at the server cimm.kpfu.ru. The models for each of the datasets and Reaction Type Control with the first neighbourhood are published on the site https://models2019.cimm.site.

## Figures and Tables

**Table 1:** Applicability domain (AD) definition methods.

Universal AD Definition Approaches		Machine Learning (ML)-Dependent AD Definition Approaches
Without Hyperparameters	With Hyperparameters	With Hyperparameters
BB	RTC_cv	GPR-AD
FC	2CC_cv	RFR_VAR
Z-1NN	1-SVM	
Leverage	Z-1NN_cv	
	Leverage_cv	

**Table 2:** Coefficient of determination (R^2^) and RMSE of predictions estimated using the nested 5-fold cross-validation (without considering the AD of models).

Regression Method		DA	S_N_2	Tautomerization	E2
RFR	R^2^	0.854	0.804	0.682	0.708
	RMSE	0.734	0.516	0.914	0.799
GPR	R^2^	0.807	0.763	0.492	0.648
	RMSE	0.845	0.568	1.156	0.876

**Table 3:** Values of four AD performance metric for four datasets for the composite AD definitions assessed using nested cross-validation with hyperparameters tuned in its inner loop.

№	AD Definition Method	Coverage				OIR				∆R^2^_AD				OD			
		S_N_2	DA	E2	Tautomerization	S_N_2	DA	E2	Tautomerization	S_N_2	DA	E2	Tautomerization	S_N_2	DA	E2	Tautomerization
ML-Dependent AD Definition Methods																	
1	RFR_VAR*/OIR	0.98	0.94	0.93	0.95	0.66	1.38	0.71	1.63	0.01	0.05	0.04	−0.05	0.56	0.73	0.68	0.73
2	GPR-AD*/OIR	0.97	0.94	0.92	0.96	0.70	0.67	0.41	1.73	0.03	0.05	0.05	0.01	0.63	0.73	0.75	0.72
3	RFR_VAR*/OD	0.79	0.83	0.81	0.90	0.51	1.22	0.57	1.74	0.07	0.10	0.10	−0.11	0.80	0.86	0.76	0.91
4	GPR-AD*/OD	0.89	0.78	0.81	0.93	0.78	0.38	0.07	1.60	0.09	0.08	0.08	−0.03	0.80	0.79	0.84	0.82
Universal AD definition methods with hyperparameters																	
5	2CC*/OIR	0.98	0.94	0.92	0.94	0.67	1.62	0.46	1.83	0.02	0.06	0.02	−0.10	0.59	0.77	0.63	0.76
6	Lev_cv*/OIR	0.98	0.94	0.75	0.96	0.61	1.39	0.15	1.59	0.01	0.05	0.02	−0.05	0.55	0.71	0.59	0.69
7	Z-1NN_cv*/OIR	0.98	0.94	0.93	0.95	0.60	1.39	0.50	1.53	0.01	0.05	0.03	−0.09	0.55	0.71	0.63	0.69
8	1-SVM*/OIR	0.98	0.94	0.86	0.86	0.57	1.34	0.35	0.79	0.01	0.05	0.03	−0.13	0.56	0.71	0.67	0.68
9	2CC*/OD	0.84	0.82	0.80	0.89	0.59	1.12	0.44	1.64	0.07	0.09	0.09	−0.12	0.82	0.83	0.74	0.87
10	Lev_cv*/OD	0.83	0.72	0.85	0.82	0.28	0.59	0.42	1.13	0.03	0.07	0.07	−0.15	0.61	0.73	0.74	0.83
11	Z-1NN_cv*/OD	0.79	0.73	0.81	0.74	0.35	0.69	0.40	0.86	0.05	0.08	0.08	−0.08	0.70	0.75	0.70	0.83
12	1-SVM*/OD	0.49	0.29	0.68	0.66	0.22	0.37	0.28	0.42	0.07	0.07	0.07	−0.21	0.69	0.62	0.72	0.67
Universal AD definition methods without hyperparameters																	
13	RTC1	0.98	0.94	0.93	0.96	0.61	1.39	0.51	1.59	0.01	0.05	0.03	−0.05	0.55	0.71	0.63	0.69
14	BB*	0.98	0.94	0.93	0.94	0.58	1.34	0.50	1.30	0.01	0.05	0.03	−0.05	0.56	0.71	0.63	0.68
15	Leverage*	0.95	0.93	0.91	0.92	0.45	1.29	0.55	1.19	0.02	0.05	0.04	−0.19	0.60	0.72	0.67	0.71
16	Z-1NN*	0.95	0.91	0.90	0.92	0.44	1.09	0.64	1.16	0.02	0.05	0.06	−0.12	0.60	0.74	0.71	0.71
“Zero models”																	
17	OZ	1.00	1.00	1.00	1.00	0	0	0	0	0.00	0.00	0.00	0.00	0.50	0.50	0.50	0.50
18	PZ	0.00	0.00	0.00	0.00	0	0	0	0	−0.81	−0.85	−0.71	−0.70	0.50	0.50	0.50	0.50
19	“Perfect AD model”	0.98	0.98	0.99	0.98	1.75	3.75	2.71	4.08	0.06	0.08	0.05	0.06	1.00	1.00	1.00	1.00

**Table 4:** Ranking of different AD definition methods.

	Data Set			
Rank	S_N_2	Tautomerization	E2	DA
1	“Perfect AD model”	“Perfect AD model”	“Perfect AD model”	“Perfect AD model”
2	GPR-AD*/OIR	RFR_VAR*/OIR	Z-1NN*	2CC*/OIR
3	2CC*/OD	GPR-AD*/OIR	RFR_VAR*/OIR	RFR_VAR*/OIR
4	GPR-AD*/OD	GPR-AD*/OD	GPR-AD*/OIR	RFR_VAR*/OD
5	RFR_VAR*/OIR	2CC*/OIR	RFR_VAR*/OD	Lev_cv*/OIR

**Table 5:** Coefficient of determination (R^2^) and RMSE of prediction for the external test set (only reactions within AD are considered).

Best-Ranked Composite Ads				
№	AD Method	R^2^	RMSE	Coverage
1	GPR-AD*/OIR	0.96	0.17	17
2	RFR_VAR*/OIR	0.66	0.64	74
3	2CC*/OIR	0.66	0.64	74
4	GPR-AD*/OD	0.96	0.17	17
5	RFR_VAR*/OD	0.80	0.39	34
6	Z-1NN_cv*/OIR	0.66	0.64	74
7	2CC*/OD	0.67	0.53	25
8	RTC1	0.66	0.64	74
9	Z-1NN_cv*/OD	0.94	0.22	17
10	BB*	0.66	0.64	73
	Without AD	0.60	0.84	100

## Supplementary Materials

The following are available online at https://www.mdpi.com/1422-0067/21/15/5542/s1, Table S1. Detailed information about the hyperparameters of AD definition approaches. Table S2. Values of four AD performance metric for all datasets for some of the individual AD definitions assessed using nested cross-validation with hyperparameters tuned in its inner loop. Tables S3–S6. Ranking of different AD definition methods for S_N_2, E2, DA, Tautomerization datasets, respectively. Table S7. Ranking of different AD definition methods for all datasets. Table S8. Coefficient of determination (R^2^) and RMSE of prediction for the external test set (only reactions within AD are considered).

## Abbreviations

AD	Applicability Domain
QSAR/QSPR	Quantitative Structure–Activity/Property Relationship
OECD	Organization of Economic Co-operation and Development
QRPR model	Quantitative Reaction–Property Relationships model
ML-dependent AD	Applicability domain definition approaches that are tied to the machine learning method. It means that the determination of whether an object belongs to the model’s applicability domain and the prediction of property value for a given object is made by the machine-learning approach.
Universal AD	Applicability domain definition approaches that are not tied to the machine learning method. In this case, predicted property is given by Random Forest Regressor and the object’s belonging to the model’s applicability domain is determined in another way.
Leverage	Universal AD definition approach which based on the distance from the test sample objects to the centre of the training set distribution.
Lev_cv	Modification of Leverage approach. Leverage does not have internal hyperparameters, Lev_cv has one—the optimal threshold that needs to be adjusted.
Z-1NN	AD is defined on the basis of distance from a test set object to the most similar training set object(s). One nearest neighbour is considered.
Z-1NN_cv	Modification of Z-1NN_cv approach. Z-1NN doesn’t have internal hyperparameters, Z-1NN_cv has one—the optimal threshold that needs to be adjusted.
1-SVM	One-Class Support Vector Machine
2CC	Two-Class Classification method
RFClf	Random Forest Classifier
BB	Bounding Box
FC	Fragment Control
RTC_cv	Reaction Type Control. It needs to add «cv» since it is necessary to select the hyperparameter of the method
RTC*	Reaction Type Control with the first neighborhood
GPR	Gaussian Process Regression
RF	Random Forest Regressor
GPR-model	If structure-reactivity modelling is performed using Gaussian Process Regression
GPR-AD	The variance in prediction by Gaussian Process as AD measure
RFR_VAR	The variance in the ensemble of predictions as AD measure
Weak consensus	If most methods suggest that the reaction falls in AD, then it is predicted as belonging to AD
Strict consensus	The reaction belongs to AD if all the methods suggest that it falls in AD
OZ	“optimistic zero model”
PZ	“pessimistic zero model”
“Perfect AD model”	Exactly predicts inliers (reactions with prediction error less than 3xRMSE on cross-validation) and outliers (the rest).
RMSE	Root-mean-square error
R^2^	Coefficient of determination
∆R^2^_AD	It is the difference between the coefficient of determination for property prediction (R^2^) for objects within AD and corresponding value for all datapoints.
OIR	It is the difference between prediction RMSE for objects outside AD (denoted as RMSE_OUT_) and within AD (denoted as RMSE_IN_).
OD	Outliers Detection
AUC_AD	Area Under the ROC Curve as ranking criterion for AD detection
CV	Cross-validation
S_N_2	Bimolecular nucleophilic substitution
DA	Diels–Alder reactions
E2	Bimolecular elimination
CGR	Condensed Graph of Reaction
