# Supplementary material for: Comprehensive Analysis of Applicability Domains of QSPR Models for Chemical Reactions
Source: Int J Mol Sci. 2020 Aug 3;21(15):5542. doi: 10.3390/ijms21155542 (PMC7432167; doi:10.3390/ijms21155542)
Supplement: Supplementary file 1 [file ijms-21-05542-s001.pdf]

Supporting Information

Comprehensive Analysis of Applicability Domains of QSPR Models for Chemical Reactions

Assima Rakhimbekova [1], Timur I. Madzhidov [1,*], Ramil I. Nugmanov [1], Timur R. Gimadiev [2], Igor I. Baskin [1,3,4] and Alexandre Varnek [2,4,*]

[1] A.M. Butlerov Institute of Chemistry, Kazan Federal University, 420008 Kazan, Russia; ARahimbekova@kpfu.ru (A.R.); RaINugmanov@kpfu.ru (R.I.N.); igbaskin@gmail.com (I.I.B.)

[2] Institute for Chemical Reaction Design and Discovery, Hokkaido University, 001-0021 Sapporo, Japan; gimadiev@icredd.hokudai.ac.jp

[3] Faculty of Physics, Moscow State University, 119234Moscow, Russia

[4] Laboratory of Chemoinformatics, UMR 7140 CNRS, University of Strasbourg, 67000 Strasbourg, France

* Correspondence: timur.madzhidov@kpfu.ru (T.I.M.); varnek@unistra.fr (A.V.)

Table S1. The values of hyperparameters for Quantitative Reaction-Property (QRPR) models and applicability domain definition methods

Table S2. Values of four AD performance metric for all data sets for some of the individual AD definitions assessed using nested cross-validation with hyperparameters tuned in its inner loop

Table S3. Ranking of different AD definition methods for SN2 data set

Table S4. Ranking of different AD definition methods for E2 data set

Table S5. Ranking of different AD definition methods for DA data set

Table S6. Ranking of different AD definition methods for Tautomerization data

Table S7. Ranking of different AD definition methods for all data sets

Table S8. Coefficient of determination (R^2) and RMSE of prediction for the external test set

Table S1. The values of hyperparameters for Quantitative Reaction-Property (QRPR) models and applicability domain definition methods

	Hyperparameters	Ranges
Random Forest Regression (RFR)	*max_features*	from 5 to 35 percentages of the total number of features (descriptors) with step = 5; logarithm and square root of the total number of features and just number of features
	n_estimators	500
Random Forest Classifier	*max_features*	from 5 to 35 percentages of the total number of features (descriptors) with step = 5; logarithm and square root of the total number of features and just number of features
	n_estimators	500
Gaussian Process Regression (GPR-AD)	*alpha*	from 10^{-8} to 10 with step = 10
	kernel	from 10^{-6} to 10^{4} with step = 10
1-SVM	*nu*	0.001, 0.01, 0.1, 0.15, ..., 0.5
	gamma	10^{-6}, 10^{-5}, ..., 10^{4}
Reaction Type Control (RTC_cv)	*R*	0, 1, 2, …, 10

Table S2. Values of four AD performance metric for all data sets for some of the individual AD definitions assessed using nested cross-validation with hyperparameters tuned in its inner loop

№	AD definition method	Coverage				OIR				ΔR^2_AD				OD			
		S_N2	DA	E2	Tautomerization	S_N2	DA	E2	Tautomerization	S_N2	DA	E2	Tautomerization	S_N2	DA	E2	Tautomerization
ML-dependent AD definition methods (Hyperparameters tuned by maximizing OIR)																	
1	RFR_VAR/OIR	0.99	0.99	0.99	0.98	0.88	1.42	2.55	2.63	0.00	0.01	0.01	-0.03	0.51	0.56	0.55	0.70
2	GRP/OIR	0.98	0.99	0.98	0.99	0.73	1.33	0.56	1.28	0.02	0.02	0.02	0.02	0.58	0.60	0.60	0.56
Universal AD definition approaches (Hyperparameters tuned by maximizing OIR)																	
3	RTC_cv/OIR	0.98	0.99	0.96	0.97	0.60	4.54	0.46	0.42	0.01	0.01	0.02	-0.01	0.55	0.53	0.57	0.49
4	2CC/OIR	0.99	0.99	0.99	0.98	0.79	2.35	-0.11	2.20	0.01	0.01	-0.01	-0.09	0.53	0.59	0.50	0.66
5	Lev_cv/OIR	0.99	1.00	0.80	0.99	-0.20	0	0.01	0.28	0.00	0.00	-0.01	-0.01	0.50	0.50	0.52	0.49
6	Z-1NN_cv/OIR	0.99	0.99	0.99	0.98	0.09	1.03	0.93	0.52	0.00	0.00	0.00	-0.03	0.50	0.50	0.55	0.49
7	1-SVM/OIR	0.99	0.99	0.92	0.89	0.55	2.25	0.27	0.25	0.00	0.01	0.01	-0.07	0.52	0.53	0.60	0.53
ML-dependent AD definition methods (Hyperparameters tuned by maximizing OD)																	
8	RFR_VAR/OD	0.80	0.84	0.85	0.91	0.49	1.24	0.59	1.74	0.07	0.10	0.10	-0.17	0.79	0.87	0.74	0.88
9	GRP/OD	0.89	0.79	0.83	0.95	0.79	0.38	0.07	1.81	0.09	0.08	0.07	-0.04	0.80	0.80	0.85	0.81
Universal AD definition methods (Hyperparameters tuned by maximizing OD)																	
10	RTC_cv/OD	0.92	0.91	0.71	0.88	0.37	1.36	0.21	1.67	0.03	0.07	0.07	-0.04	0.60	0.77	0.62	0.95
11	2CC/OD	0.85	0.84	0.84	0.91	0.57	1.14	0.42	1.63	0.06	0.09	0.09	-0.17	0.80	0.84	0.71	0.84
12	Lev_cv/OD	0.83	0.73	0.89	0.82	0.28	0.50	0.46	1.14	0.02	0.06	0.06	-0.16	0.61	0.71	0.71	0.83
13	Z-1NN_cv/OD	0.79	0.73	0.83	0.74	0.35	0.69	0.42	0.87	0.05	0.08	0.08	-0.07	0.70	0.75	0.70	0.83
14	1-SVM/OD	0.49	0.29	0.71	0.68	0.22	0.37	0.26	0.29	0.07	0.07	0.07	-0.21	0.69	0.62	0.69	0.63
Universal AD definition methods without hyperparameters																	
15	FC	0.99	0.99	0.98	0.97	0.65	2.14	0.67	1.15	0.01	0.02	0.01	0.01	0.53	0.54	0.56	0.53
16	BB	0.99	0.99	0.99	0.98	0.41	2.48	0.72	0.62	0.00	0.01	0.01	-0.02	0.51	0.53	0.54	0.53
17	Leverage	0.96	0.97	0.97	0.92	0.43	1.45	0.85	1.27	0.02	0.03	0.03	-0.19	0.58	0.59	0.65	0.72
18	Z-1NN	0.95	0.94	0.94	0.93	0.39	0.94	0.79	1.07	0.02	0.03	0.04	-0.15	0.57	0.62	0.69	0.67

"Zero models"																	
19	OZ	1.00	1.00	1.00	1.00	0	0	0	0	0.00	0.00	0.00	0.00	0.50	0.50	0.50	0.50
20	PZ	0.00	0.00	0.00	0.00	0	0	0	0	-0.81	-0.85	-0.71	-0.70	0.50	0.50	0.50	0.50
21	“Perfect AD Model”	0.98	0.98	0.99	0.98	1.75	3.75	2.71	4.08	0.06	0.08	0.05	0.06	1.00	1.00	1.00	1.00

Table S3. Ranking of different AD definition methods for S_N2 data set[1]

№	AD definition approaches	Metrics					Weights					penalty	rank
		Coverage	OIR	ΔR^2_AD	OD	reaction of non-native type	Coverage	OIR	ΔR^2_AD	OD	reaction of non-native type		
1	"Perfect AD Model"	0.98	1.75	0.06	1.00	0	0	0	0	0	0	0	1
2	GPR-AD*/OIR	0.97	0.70	0.03	0.63	0	0	0	0	0	0	0	1
3	2CC*/OD	0.84	0.59	0.07	0.82	0	1	0	0	0	0	1	3
4	GPR-AD*/OD	0.89	0.78	0.09	0.80	0	1	0	0	0	0	1	3
5	RFR_VAR*/OIR	0.98	0.66	0.01	0.56	0	0	0	1	1	0	2	5
6	2CC*/OIR	0.98	0.67	0.02	0.59	0	0	0	1	1	0	2	5
7	Lev_cv*/OIR	0.98	0.61	0.01	0.55	0	0	0	1	1	0	2	5
8	Z1NN_cv*/OIR	0.98	0.60	0.01	0.55	0	0	0	1	1	0	2	5
9	RFR_VAR*/OD	0.79	0.51	0.07	0.80	0	1	1	0	0	0	2	5
10	Lev_cv*/OD	0.83	0.28	0.03	0.61	0	1	1	0	0	0	2	5
11	Z1NN_cv*/OD	0.79	0.35	0.05	0.70	0	1	1	0	0	0	2	5
12	1-SVM*/OD	0.49	0.22	0.07	0.69	0	1	1	0	0	0	2	5
13	RTC*	0.98	0.61	0.01	0.55	0	0	0	1	1	0	2	5
14	BB*	0.98	0.58	0.01	0.56	0	0	0	1	1	0	2	5
15	Leverage*	0.95	0.45	0.02	0.60	0	1	1	0	0	0	2	5
16	Z1NN*	0.95	0.44	0.02	0.60	0	1	1	0	0	0	2	5
17	1-SVM*/OIR	0.98	0.57	0.01	0.56	0	0	1	1	1	0	3	17
18	OZ	1.00	-0.51	0.00	0.50	0	0	1	1	1	0	3	17
19	PZ	0.00	0.51	-0.81	0.50	0	1	1	1	1	0	4	19

[1] * means combination with Reaction Type Control with the first neighbourhood (*R*=1, RTC1)

Table S4. Ranking of different AD definition methods for E2 data set[2]

№	AD definition approaches	Metrics					Weights					penalty	rank
		Coverage	OIR	ΔR^2_AD	OD	reaction of non-native type	Coverage	OIR	ΔR^2_AD	OD	reaction of non-native type		
1	“Perfect AD Model”	0.99	2.71	0.05	1.00	0	0	0	0	0	0	0	1
2	Z1NN*	0.90	0.64	0.06	0.71	0	0	0	0	0	0	0	1
3	RFR_VAR*/OIR	0.93	0.71	0.04	0.68	0	0	0	1	0	0	1	3
4	GPR-AD*/OIR	0.92	0.41	0.05	0.75	0	0	1	0	0	0	1	3
5	RFR_VAR*/OD	0.81	0.57	0.10	0.76	0	1	0	0	0	0	1	3
6	2CC*/OIR	0.92	0.46	0.02	0.63	0	0	0	1	1	0	2	6
7	Z1NN_cv*/OIR	0.93	0.50	0.03	0.63	0	0	0	1	1	0	2	6
8	2CC*/OD	0.80	0.44	0.09	0.74	0	1	1	0	0	0	2	6
9	Lev_cv*/OD	0.85	0.42	0.07	0.74	0	1	1	0	0	0	2	6
10	Z1NN_cv*/OD	0.81	0.40	0.08	0.70	0	1	1	0	0	0	2	6
11	1-SVM*/OD	0.68	0.28	0.07	0.72	0	1	1	0	0	0	2	6
12	GPR-AD*/OD	0.81	0.07	0.08	0.84	0	1	1	0	0	0	2	6
13	RTC*	0.93	0.51	0.03	0.63	0	0	0	1	1	0	2	6
14	BB*	0.93	0.50	0.03	0.63	0	0	0	1	1	0	2	6
15	Leverage*	0.91	0.55	0.04	0.67	0	0	0	1	1	0	2	6
16	OZ	1.00	-0.80	0.00	0.50	0	0	1	1	1	0	3	16
17	PZ	0.00	0.80	-0.71	0.50	0	1	0	1	1	0	3	16
18	Lev_cv*/OIR	0.75	0.15	0.02	0.59	0	1	1	1	1	0	4	19
19	1-SVM*/OIR	0.86	0.35	0.03	0.67	0	1	1	1	1	0	4	19

[2] * means combination with Reaction Type Control with the first neighbourhood (*R*=1, RTC1)

Table S5. Ranking of different AD definition methods for DA data set[3]

№	AD definition approaches	Metrics					Weights					penalty	rank
		Coverage	OIR	ΔR^2_AD	OD	reaction of non-native type	Coverage	OIR	ΔR^2_AD	OD	reaction of non-native type		
1	"Perfect AD Model"	0.98	3.75	0.08	1.00	0	0	0	0	0	0	0	1
2	2CC*/OIR	0.94	1.62	0.06	0.77	0	0	0	0	0	0	0	1
3	RFR_VAR*/OIR	0.94	1.38	0.05	0.73	0	0	0	1	0	0	1	3
4	RFR_VAR*/OD	0.83	1.22	0.10	0.86	0	1	0	0	0	0	1	3
5	Lev_cv*/OIR	0.94	1.39	0.05	0.71	0	0	0	1	1	0	2	5
6	Z1NN_cv*/OIR	0.94	1.39	0.05	0.71	0	0	0	1	1	0	2	5
7	1-SVM*/OIR	0.94	1.34	0.05	0.71	0	0	0	1	1	0	2	5
8	GPR-AD*/OIR	0.94	0.67	0.05	0.73	0	0	1	1	0	0	2	5
9	2CC*/OD	0.82	1.12	0.09	0.83	0	1	1	0	0	0	2	5
10	Lev_cv*/OD	0.72	0.59	0.07	0.73	0	1	1	0	0	0	2	5
11	Z1NN_cv*/OD	0.73	0.69	0.08	0.75	0	1	1	0	0	0	2	5
12	GPR-AD*/OD	0.78	0.38	0.08	0.79	0	1	1	0	0	0	2	5
13	RTC*	0.94	1.39	0.05	0.71	0	0	0	1	1	0	2	5
14	BB*	0.94	1.34	0.05	0.71	0	0	0	1	1	0	2	5
15	Leverage*	0.93	1.29	0.05	0.72	0	1	0	0	1	0	2	5
16	Z1NN*	0.91	1.09	0.05	0.74	0	1	1	0	0	0	2	5
17	1-SVM*/OD	0.29	0.37	0.07	0.62	0	1	1	0	1	0	3	17
18	OZ	1.00	-0.73	0.00	0.50	0	0	1	1	1	0	3	17
19	PZ	0.00	0.73	-0.85	0.50	0	1	1	1	1	0	4	19

[3] * means combination with Reaction Type Control with the first neighbourhood (*R*=1, RTC1)

Table S6. Ranking of different AD definition methods for Tautomerization data[4]

№	AD definition approaches	Coverage	OIR	ΔR^2_AD	OD	reaction of non-native type	Coverage	OIR	ΔR^2_AD	OD	reaction of non-native type	penalty	rank
1	“Perfect AD Model”	0.98	4.08	0.06	1.00	0	0	0	0	0	0	0	1
2	RFR_VAR*/OIR	0.95	1.63	-0.05	0.73	0	0	0	0	0	0	0	1
3	GPR-AD*/OIR	0.96	1.73	0.01	0.72	0	0	0	0	0	0	0	1
4	GPR-AD*/OD	0.93	1.60	-0.03	0.82	0	0	0	0	0	0	0	1
5	2CC*/OIR	0.94	1.83	-0.10	0.76	0	0	0	1	0	0	1	5
6	Lev_cv*/OIR	0.96	1.59	-0.05	0.69	0	0	0	0	1	0	1	5
7	Z1NN_cv*/OIR	0.95	1.53	-0.09	0.69	0	0	0	0	1	0	1	5
8	RTC*	0.96	1.59	-0.05	0.69	0	0	0	0	1	0	1	5
9	RFR_VAR*/OD	0.90	1.74	-0.11	0.91	0	1	0	1	0	0	2	9
10	2CC*/OD	0.89	1.64	-0.12	0.87	0	1	0	1	0	0	2	9
11	Z1NN_cv*/OD	0.74	0.86	-0.08	0.83	0	1	1	0	0	0	2	9
12	BB*	0.94	1.30	-0.05	0.68	0	0	1	0	1	0	2	9
13	OZ	1.00	-0.89	0.00	0.50	0	0	1	0	1	0	2	9
14	Lev_cv*/OD	0.82	1.13	-0.15	0.83	0	1	1	1	0	0	3	14
15	1-SVM*/OIR	0.86	0.79	-0.13	0.68	0	1	1	1	1	0	4	15
16	1-SVM*/OD	0.66	0.42	-0.21	0.67	0	1	1	1	1	0	4	15
17	Leverage*	0.92	1.19	-0.19	0.71	0	1	1	1	1	0	4	15
18	Z1NN*	0.92	1.16	-0.12	0.71	0	1	1	1	1	0	4	15
19	PZ	0.00	0.89	-0.70	0.50	0	1	1	1	1	0	4	15

[4] * means combination with Reaction Type Control with the first neighbourhood (R=1, RTC1)

Table S7. Ranking of different AD definition methods for all data sets[5]

№	AD definition approach	Coverage				OIR				ΔR^2_AD				OD				reaction of non-native type				penalty	rank
		S_N2	DA	E2	T	S_N2	DA	E2	T	S_N2	DA	E2	T	S_N2	DA	E2	T	S_N2	DA	E2	T		
1	“Perfect AD Model”	0	0	0	0	0	0	0	0	0	0	0	0	0	0	0	0	0	0	0	0	0	1
2	GPR-AD*/OIR	0	0	0	0	0	1	1	0	0	1	0	0	0	0	0	0	0	0	0	0	3	2
3	RFR_VAR*/OIR	0	0	0	0	0	0	0	0	1	1	1	0	1	0	0	0	0	0	0	0	4	3
4	2CC*/OIR	0	0	0	0	0	0	0	0	1	0	1	1	1	0	1	0	0	0	0	0	5	4
5	GPR-AD*/OD	1	1	1	0	0	1	1	0	0	0	0	0	0	0	0	0	0	0	0	0	5	4
6	RFR_VAR*/OD	1	1	1	1	1	0	0	0	0	0	0	1	0	0	0	0	0	0	0	0	6	6
7	Z1NN_cv*/OIR	0	0	0	0	0	0	0	0	1	1	1	0	1	1	1	1	0	0	0	0	7	7
8	2CC*/OD	1	1	1	1	0	1	1	0	0	0	0	1	0	0	0	0	0	0	0	0	7	7
9	RTC*	0	0	0	0	0	0	0	0	1	1	1	0	1	1	1	1	0	0	0	0	7	7
10	Z1NN_cv*/OD	1	1	1	1	1	1	1	1	0	0	0	0	0	0	0	0	0	0	0	0	8	10
11	BB*	0	0	0	0	0	0	0	1	1	1	1	0	1	1	1	1	0	0	0	0	8	10
12	Z1NN*	1	1	0	1	1	1	0	1	0	0	0	1	0	0	0	1	0	0	0	0	8	10
13	Lev_cv*/OIR	0	0	1	0	0	0	1	0	1	1	1	0	1	1	1	1	0	0	0	0	9	13
14	Lev_cv*/OD	1	1	1	1	1	1	1	1	0	0	0	1	0	0	0	0	0	0	0	0	9	13
15	Leverage*	1	1	0	1	1	0	0	1	0	0	1	1	0	1	1	1	0	0	0	0	10	15
16	1-SVM*/OD	1	1	1	1	1	1	1	1	0	0	0	1	0	1	0	1	0	0	0	0	11	16
17	OZ	0	0	0	0	1	1	1	1	1	1	1	0	1	1	1	1	0	0	0	0	11	16
18	1-SVM*/OIR	0	0	1	1	1	0	1	1	1	1	1	1	1	1	1	1	0	0	0	0	13	18
19	PZ	1	1	1	1	1	1	0	1	1	1	1	1	1	1	1	1	0	0	0	0	15	19

[5] * means combination with Reaction Type Control with the first neighbourhood (*R*=1, RTC1)

Table S8. Coefficient of determination (R^2) and RMSE of prediction for the external test set (only reactions within AD are considered)[6]

Ranked composite ADs					Individual ADs			
№	AD method	R^2	RMSE	Coverage	AD method	R^2	RMSE	Coverage
2	RFR_VAR*/OIR	0.66	0.64	74	RFR_VAR/OIR	0.60	0.84	100
3	GPR-AD*/OIR	0.96	0.17	17	GPR-AD/OIR	0.96	0.17	17
4	2CC*/OIR	0.66	0.64	74	2CC/OIR	0.60	0.84	100
5	GPR-AD*/OD	0.96	0.17	17	GPR-AD/OD	0.96	0.17	17
6	RFR_VAR*/OD	0.80	0.39	34	RFR_VAR/OD	0.80	0.40	34
7	Z1NN_cv*/OIR	0.66	0.64	74	Z1NN_cv/OIR	0.60	0.84	100
8	2CC*/OD	0.67	0.53	25	2CC/OD	0.71	0.62	36
9	RTC1	0.66	0.64	74				
10	Z1NN_cv*/OD	0.94	0.22	17	Z1NN_cv/OD	0.94	0.22	17
11	BB*	0.66	0.64	73	BB	0.61	0,84	99
12	Z1NN*	0.92	0.25	18	Z1NN	0.92	0.25	18
13	Lev_cv*/OIR	0.66	0.64	74	Lev_cv/OIR	0.60	0.84	100
14	Lev_cv*/OD	0.83	0.36	21	Lev_cv/OD	0.83	0.36	21
15	Leverage*	0.80	0.38	22	Leverage	0.80	0.38	22
16	1-SVM*/OD	0.00	0.00	0	1-SVM/OD	0.00	0.00	0
17	1-SVM*/OIR	0.66	0.64	74	1-SVM/OIR	0.60	0.84	100

[6] * means combination with Reaction Type Control with the first neighbourhood (*R*=1, RTC1)
